# Determinants of Health‐Related Quality of Life in Children and Adolescents With Type 1 Diabetes: A Cross‐Sectional Study

**DOI:** 10.1002/hsr2.73143

**Published:** 2026-08-30

**Authors:** Solmaz Heidari, Majid Aminzadeh, Ronak Badiei, Mahtab Tamimi, Shooka Mohammadi

**Affiliations:** ^1^ Department of Pediatrics, Faculty of Medicine Ahvaz Jundishapur University of Medical Sciences Ahvaz Iran

**Keywords:** adolescents, children, health‐related quality of life, PedsQL, type 1 diabetes

## Abstract

**Background and Aim:**

Type 1 diabetes mellitus (T1DM) is a chronic condition that affects emotional, physical, social, and academic functioning of children and adolescents. Assessing health‐related quality of life (HRQoL) provides insight into the broader impact of T1DM. This study evaluated HRQoL among pediatric patients with T1DM in southwest Iran using reports from children and parents.

**Methods:**

This cross‐sectional study included 234 children aged 5–16 years with T1DM and their parents at Abuzar Hospital (Ahvaz, Iran). HRQoL was assessed using the Pediatric Quality of Life Inventory (PedsQL) version 4.0 completed by both children and parents, and the PedsQL 3.2 Diabetes Module. Demographic, clinical, and lifestyle characteristics were collected.

**Results:**

Children reported the highest scores for physical functioning and the lowest scores for school functioning. Parents rated their children's HRQoL significantly lower than children did across all domains (*p* < 0.001). Boys had significantly higher HRQoL than girls in all generic domains except school functioning and in all diabetes‐specific domains (*p* < 0.05). Children reported higher HRQoL than adolescents, with significant differences in most domains except physical functioning (*p* < 0.05). Common unhealthy behaviors included physical inactivity (31.6%), breakfast skipping (31.2%), and weekly fast‐food consumption (43.2%). Only 26.5% of participants consistently practiced carbohydrate counting. Multivariable linear regression analysis showed that male sex, regular physical activity, and consistent carbohydrate counting were independently associated with higher total PedsQL scores (*p* < 0.05).

**Conclusion:**

Pediatric patients with T1DM in this study had the lowest HRQoL scores in school functioning. Differences between children's and parents' reports suggest that parents may perceive a greater impact of diabetes than children. Lower HRQoL among girls highlights the need for additional psychosocial support. Routine HRQoL assessment, psychosocial support, regular physical activity, carbohydrate counting education, and family‐centered care may improve overall well‐being in these patients.

## 1 Introduction

Type 1 diabetes mellitus (T1DM) is an autoimmune disorder characterized by immune‐mediated destruction of pancreatic β‐cells, resulting in absolute insulin deficiency and chronic hyperglycemia [1]. It represents a growing global health concern among children and adolescents, with increasing incidence reported worldwide [2, 3, 4, 5, 6, 7]. The prevalence of T1DM has increased worldwide in recent decades [8, 9]. In Iran, national and regional data also indicate a rising prevalence of T1DM, highlighting the need for comprehensive management strategies that extend beyond glycemic control [10, 11].

Children and adolescents with T1DM require frequent blood glucose monitoring, lifelong insulin therapy, and continuous adherence to dietary and lifestyle recommendations [12, 13]. These daily demands can affect emotional well‐being, physical functioning, social interactions, and school performance [14, 15]. In addition to these treatment‐related challenges, children with T1DM may experience anxiety, fear of hypoglycemia, and psychological stress, which can further affect their overall well‐being and adaptation to the disease [15, 16, 17, 18].

Health‐related quality of life (HRQoL) is an important outcome in children and adolescents with chronic diseases, including T1DM [19, 20, 21, 22]. It reflects an individual's perception of physical, emotional, social, and school functioning and provides valuable information beyond clinical measures such as hemoglobin A1c (HbA1c) [14, 17, 22]. Previous studies have shown that children with T1DM often report moderate to impaired HRQoL, particularly in the emotional and school domains [20, 22, 23, 24, 25]. Children's perceptions of their HRQoL may also differ from those of their parents, highlighting the importance of assessing both perspectives [26, 27, 28].

Assessing HRQoL is increasingly recognized as an essential component of comprehensive diabetes care [29]. Incorporating patient‐reported outcomes provides a more holistic understanding of the burden of T1DM from both the child's and family's perspectives. This approach aligns with current models of chronic disease management that emphasize patient‐centered and psychosocial care [15, 30]. Routine assessment of HRQoL may help clinicians identify psychosocial concerns early, improve communication with patients and families, and guide individualized interventions to support treatment adherence and long‐term outcomes [31, 32]. Several factors have been associated with HRQoL in pediatric T1DM, including age, sex, disease duration, glycemic control, diabetes‐related complications, parental education, and socioeconomic status [24, 25, 33]. Several studies have also reported lower HRQoL among girls than boys [24, 34]. In addition, cultural context and access to healthcare resources may influence HRQoL, emphasizing the importance of region‐specific studies [24, 35].

Despite the increasing prevalence of T1DM in Iran, evidence on HRQoL among affected children and adolescents remains limited, particularly in the southwest region of the country. To our knowledge, no previous study has specifically evaluated HRQoL among children and adolescents with T1DM in Ahvaz, southwest Iran. Therefore, this study assessed HRQoL in pediatric patients with T1DM using both children's self‐reports and parents' proxy reports. Understanding HRQoL in these patients may help identify psychosocial needs and support the development of targeted, patient‐centered care strategies.

## 2 Materials and Methods

### 2.1 Study Design and Setting

This cross‐sectional study was conducted at Abuzar Hospital (Ahvaz, Iran) from April 2024 to April 2026 and included children with T1DM and their parents or guardians. Eligible participants were recruited during the study period. HRQoL assessments and data collection were performed at the time of enrollment.

### 2.2 Participants and Sampling

Children aged 5 to 16 years with a confirmed diagnosis of T1DM and no additional comorbidities or underlying diseases were eligible for inclusion. Consecutive sampling was employed. All eligible children with T1DM who were referred to the hospital during the study period, along with their parents, were invited to participate. Basic clinical and demographic information, including sex, age, and medical history, was collected using a structured data collection checklist. Before enrollment, all parents or legal guardians provided written informed consent.

### 2.3 Data Collection Instrument

HRQoL was evaluated using the Pediatric Quality of Life Inventory (PedsQL) version 4.0, a widely used generic instrument designed to assess HRQoL in children. The PedsQL 4.0 has demonstrated strong psychometric properties [17]. The questionnaire has also been validated in the Persian language, confirming its reliability and cultural appropriateness for use in Iranian children [36]. The questionnaire included 23 items across four domains: social (5 items), emotional (5 items), physical (8 items), and school functioning (5 items). Items assessed the frequency of problems experienced during the past month. Responses were scored and transformed to a 0–100 scale, with higher scores indicating better quality of life. Domain scores were calculated as the mean of their respective items. The physical health summary score was derived from the physical functioning domain, while the psychosocial health summary score was calculated as the mean of the emotional, social, and school functioning domains [17].

The PedsQL 3.2 Diabetes Module was also used as a diabetes‐specific instrument to assess HRQoL in pediatric patients with T1DM [37]. Persian versions of the PedsQL generic core scales and diabetes‐specific module have demonstrated acceptable reliability and validity among Iranian children and adolescents with T1DM [38]; therefore, validated Persian versions of these instruments were used in the present study. The module consists of 33 items across five domains: treatment adherence (6 items), treatment barriers (5 items), diabetes‐related worry (3 items), diabetes symptoms (15 items), and diabetes‐related communication (4 items). Responses were linearly transformed to a 0–100 scale, with higher scores indicating better HRQoL. Domain scores were calculated as the mean of the items within each domain [37]. Both children and their parents completed the PedsQL 4.0 Generic Core Scales, while the PedsQL 3.2 Diabetes Module was completed only by the children.

Weight and height of the patients were measured using standard protocols. BMI was calculated and converted to age‐ and sex‐specific Z‐scores. Weight status was classified according to WHO criteria. HbA1c levels were extracted from medical records as an indicator of glycemic control. In addition, some questions were asked regarding physical activity (PA) and dietary habits.

### 2.4 Statistical Analysis

Statistical analyzes were performed using SPSS version 24. The distribution of continuous variables was assessed using the Kolmogorov–Smirnov test. Continuous variables were presented as mean ± standard deviation (SD), whereas categorical variables were displayed as frequencies and percentages. Group comparisons were performed using independent and paired *t*‐tests for continuous variables and the chi‐square or Fisher's exact test for categorical variables. Multiple linear regression analysis was performed to identify factors associated with total PedsQL scores. The total PedsQL score was used as the dependent variable. Variables included in the multivariable model were selected a priori based on clinical relevance and previous literature. The results are presented as unstandardized regression coefficients (B) with 95% confidence intervals (CIs). Model fit was assessed using the coefficient of determination (R^2^), adjusted R^2^, and the F‐test (for overall model significance). All statistical tests were two‐sided, and a *p*‐value < 0.05 was considered statistically significant.

## 3 Results

A total of 234 adolescents and children with T1DM were enrolled in this study, including 106 boys (45.3%) and 128 girls (54.7%). Most participants were 11–16 years old (67.5%), and diabetes onset most commonly occurred between ages 6 and 10 (67.9%). Overall, 44.4% of participants reported a family history of diabetes, and 65.4% lived in urban areas. More than half of the mothers and nearly half of the fathers had secondary education or below (Table 1).

**Table 1:** Clinical and demographic characteristics of pediatric patients with T1DM (*n* = 234).

Characteristics		*n* (%)
Age group (years)	5–10	76 (32.5)
	11–16	158 (67.5)
Age at diabetes onset (years)	1–5	70 (29.9)
	6–10	159 (67.9)
	11–12	5 (2.1)
Family history of diabetes	Yes	104 (44.4)
	No	130 (55.6)
Living area	Urban	153 (65.4)
	Rural	81 (34.6)
Mother's education	Illiterate	51 (21.8)
	Secondary or below	84 (35.9)
	High school diploma	71 (30.3)
	Bachelor's degree	25 (10.7)
	Postgraduate	3 (1.3)
Fathers' education	Illiterate	16 (6.8)
	Secondary or below	88 (37.5)
	High school diploma	83 (35.5)
	Bachelor's degree	43 (18.4)
	Postgraduate	4 (1.7)

Demographic, clinical, and lifestyle characteristics according to gender are presented in Table 2. There were no significant differences between boys and girls in age, BMI Z‐score, duration of diabetes, insulin regimen, HbA1c levels, or age at diabetes onset (*p* > 0.05). The mean age was approximately 11.6 years in both groups, while the mean HbA1c was 9.79% in boys and 10.22% in girls. Most participants had a normal weight, with similar proportions in boys (63.2%) and girls (62.5%). The prevalence of overweight, obesity, and underweight was also comparable between the two groups (*p* > 0.05). Lifestyle behaviors were generally comparable between boys and girls. Breakfast skipping (≥ 3 times/week) was reported by 31.2% of participants, weekly consumption of sugar‐sweetened beverages by 21.8%, and weekly fast‐food consumption by 43.2%. Only 25.2% of participants met the recommended fruit and vegetable intake. Consistent carbohydrate counting was reported by only 26.5% of participants, whereas 34.6% reported never practicing it. Approximately 29% of participants engaged in daily PA, while almost 40% reported weekly PA. A considerable proportion reported no regular PA, with a slightly higher prevalence among girls; however, the difference was not statistically significant (*p* > 0.05).

**Table 2:** Demographic, clinical, and lifestyle characteristics of patients with T1DM according to gender (*n* = 234).

		Mean ± SD		
Characteristics		Boys (*n* = 106)	Girls (*n *= 128)	*p* value
Age (years)		11.61 ± 3.36	11.66 ± 3.33	> 0.05
BMI Z‐score		0.42 ± 1.10	0.38 ± 1.05	> 0.05
HbA1c (%)		9.79 ± 1.21	10.22 ± 1.54	> 0.05
Duration of diabetes (years)		4.74 ± 2.31	4.96 ± 2.45	> 0.05
Age at diabetes onset (years)		6.87 ± 1.89	6.49 ± 2.03	> 0.05
		***n* (%)**		
Duration of diabetes (years)	< 1	12 (11.3)	15 (11.7)	> 0.05
	1–5	48 (45.3)	56 (43.8)	
	> 5	46 (43.4)	57 (44.5)	
Insulin regimen	Multiple daily injections	82 (77.4)	101 (78.9)	> 0.05
	Insulin pump	24 (22.6)	27 (21.1)	
Weight status (BMI Z‐score)	Underweight (< −2)	6 (5.7)	7 (5.5)	> 0.05
	Normal weight (−2 to +1)	67 (63.2)	80 (62.5)	
	Overweight (+1 to +2)	20 (18.9)	26 (20.3)	
	Obese (> +2)	13 (12.3)	15 (11.7)	
Eating habits				
Breakfast skipping (≥ 3 times/week)		31 (29.2)	42 (32.8)	> 0.05
Sugar‐sweetened beverages (≥ 1 time/week)		20 (18.9)	31 (24.2)	> 0.05
Fast food consumption (1–2 times/week)		44 (41.5)	57 (44.5)	> 0.05
Adequate fruit and vegetable intake (≥ 5 servings/day)		31 (29.2)	28 (21.9)	> 0.05
Carbohydrate counting	Always	30 (28.3)	32 (25.0)	> 0.05
	Sometimes	41 (38.7)	50 (39.1)	
	Never	35 (33.0)	46 (35.9)	
Physical activity	Daily	34 (32.1)	33 (25.8)	> 0.05
	Weekly	42 (39.6)	51 (39.8)	
	None	30 (28.3)	44 (34.4)	

Abbreviations: BMI, body mass index; HbA1c, hemoglobin A1_C_.

Mean HRQoL scores assessed using the generic PedsQL are presented in Table 3. Based on children's self‐reports, the highest score was for physical functioning, whereas the lowest was for school functioning. Parents reported significantly lower scores than children across all HRQoL domains and summary scores, including physical, emotional, social, and school functioning, physical health, psychosocial health, and overall HRQoL (*p* < 0.001).

**Table 3:** HRQoL of pediatric patients with T1DM from children's and parents' perspectives.

	Mean ± SD		
Domains	Children (*n* = 234)	Parents (*n* = 234)	*p* value
Physical functioning	60.55 ± 17.08	57.23 ± 16.92	< 0.001
Emotional functioning	48.39 ± 13.22	43.07 ± 21.92	< 0.001
Social functioning	59.29 ± 15.60	51.73 ± 22.31	< 0.001
School functioning	46.70 ± 13.99	40.01 ± 11.81	< 0.001
Physical health summary score	60.55 ± 17.08	57.23 ± 16.92	< 0.001
Psychosocial health summary score	51.46 ± 11.51	44.93 ± 15.86	< 0.001
Total PedsQL 4.0 score	53.73 ± 11.49	48.01 ± 14.35	< 0.001

Abbreviation: PedsQL, pediatric quality of life inventory.

Gender‐based differences in HRQoL scores are presented in Table 4. Boys reported significantly higher scores than girls in physical, emotional, and social functioning, as well as in the physical and psychosocial health summary scores and overall HRQoL (*p* < 0.05). No significant difference was observed in school functioning (*p* > 0.05). In the diabetes module, boys also reported significantly higher scores than girls across all diabetes‐specific domains (treatment barriers, diabetes symptoms, diabetes‐related worry, diabetes‐related communication, and treatment adherence) (*p* < 0.05).

**Table 4:** HRQoL of pediatric patients with T1DM according to gender (*n* = 234).

	Mean ± SD		
Domains	Boys (*n* = 106)	Girls (*n* = 128)	*p* value
PedsQL 4.0			
Physical functioning	64.58 ± 16.35	57.21 ± 17.55	< 0.001
Emotional functioning	51.19 ± 12.80	46.09 ± 13.30	< 0.01
Social functioning	62.43 ± 14.60	56.70 ± 16.10	< 0.05
School functioning	47.50 ± 13.50	46.04 ± 14.30	> 0.05
Physical health summary score	64.58 ± 16.35	57.21 ± 17.55	< 0.001
Psychosocial health summary score	53.71 ± 11.40	49.61 ± 11.50	< 0.05
Total PedsQL 4.0 score	56.40 ± 11.20	51.52 ± 11.70	< 0.001
PedsQL 3.2 diabetes module			
Treatment barriers	57.05 ± 12.65	51.39 ± 14.25	< 0.01
Diabetes symptoms	59.99 ± 15.35	52.85 ± 12.85	< 0.001
Diabetes‐related worry	58.24 ± 12.15	53.22 ± 20.75	< 0.05
Diabetes‐related communication	66.56 ± 18.25	59.77 ± 17.45	< 0.01
Treatment adherence	69.02 ± 16.35	63.43 ± 14.25	< 0.01

Abbreviation: PedsQL, pediatric quality of life inventory.

HRQoL scores according to age group are presented in Table 5. Children aged 5–10 years generally reported higher scores than adolescents aged 11–16 years across the generic HRQoL domains. Significant differences were observed in emotional, social, and school functioning, as well as in the psychosocial health summary and overall HRQoL, with higher scores among younger children (*p* < 0.05). In contrast, physical functioning and the physical health summary did not differ significantly between age groups (*p* > 0.05). In the diabetes module, no significant differences were observed between children and adolescents (*p* > 0.05).

**Table 5:** HRQoL of pediatric patients with T1DM according to age group (*n* = 234).

	Mean ± SD		
Domains	Children (5–10 years) (*n* = 76)	Adolescents (11–16 years) (*n* = 158)	*p* value
PedsQL 4.0			
Physical functioning	62.67 ± 16.25	59.53 ± 17.50	> 0.05
Emotional functioning	50.89 ± 12.70	47.20 ± 13.40	< 0.05
Social functioning	62.40 ± 14.90	57.80 ± 15.80	< 0.05
School functioning	50.44 ± 13.40	44.90 ± 14.10	< 0.01
Physical health summary score	62.67 ± 16.25	59.53 ± 17.50	> 0.05
Psychosocial health summary score	54.58 ± 10.80	49.97 ± 11.30	< 0.01
Total PedsQL 4.0 score	56.71 ± 11.40	52.30 ± 11.60	< 0.01
PedsQL 3.2 diabetes module			
Treatment barriers	55.70 ± 13.80	53.10 ± 14.20	> 0.05
Diabetes symptoms	58.10 ± 14.00	55.10 ± 14.60	> 0.05
Diabetes‐related worry	58.00 ± 16.90	54.30 ± 16.70	> 0.05
Diabetes‐related communication	65.00 ± 17.50	61.80 ± 18.00	> 0.05
Treatment adherence	67.95 ± 15.00	65.00 ± 15.50	> 0.05

Abbreviation: PedsQL, pediatric quality of lglife inventory.

In the multivariable linear regression model, male sex (B = 4.82, 95% CI: 1.82–7.82, *p* < 0.01), regular PA (B = 5.13, 95% CI: 1.40–8.86, *p* < 0.01), and consistent carbohydrate counting (B = 4.95, 95% CI: 1.44–8.46, *p* < 0.01) were independently associated with higher total PedsQL scores (Table 6). However, HbA1c, age, BMI Z‐score, breakfast skipping, weekly fast‐food consumption, maternal education, and living area were not significantly associated with total PedsQL scores (*p* > 0.05). The regression model was statistically significant (F = 4.67, *p* < 0.001) and explained 17% of the variance in total PedsQL scores (R^2^ = 0.17; adjusted R^2^ = 0.14).

**Table 6:** Multiple linear regression analysis of factors associated with total PedsQL score in pediatric patients with T1DM.

Variables	B (95% CI)	*p* value
Sex (male vs female)	4.82 (1.82 to 7.82)	< 0.01
Regular PA (yes vs no)	5.13 (1.40 to 8.86)	< 0.01
Carbohydrate counting (consistent vs inconsistent)	4.95 (1.44 to 8.46)	< 0.01
HbA1c (%)	−0.85 (−1.88 to 0.18)	> 0.05
Age (years)	−0.41 (−0.84 to 0.02)	> 0.05
BMI Z‐score	0.28 (−0.92 to 1.48)	> 0.05
Breakfast skipping (yes vs no)	−1.02 (−3.88 to 1.84)	> 0.05
Weekly fast‐food consumption (yes vs rarely)	−0.89 (−3.61 to 1.83)	> 0.05
Maternal education (≥ high school vs < high school)	1.15 (−1.26 to 3.56)	> 0.05
Living area (urban vs rural)	0.78 (−2.20 to 3.76)	> 0.05

*Note*: The model was adjusted for all variables listed in the table.

Abbreviations: B, unstandardized regression coefficients; BMI, body mass index; CI, confidence interval; PA, physical activity.

## 4 Discussion

The present study evaluated HRQoL among adolescents and children with T1DM in southwest Iran using both children's self‐reports and parents' proxy reports. The findings highlight differences between children's and parents' perspectives, as well as the influence of gender, lifestyle behaviors, and psychosocial factors on HRQoL in pediatric T1DM.

In this study, adolescents reported lower HRQoL than younger children across several generic HRQoL domains. Adolescence represents an important developmental period in diabetes care because increasing independence, greater responsibility for self‐management, and psychosocial changes may influence disease‐related experiences and quality of life [3, 8, 9]. Children aged 5–10 years reported significantly higher HRQoL than adolescents aged 11–16 years in emotional, social, and school functioning, as well as in the psychosocial health summary score and total HRQoL score. In contrast, physical functioning and physical health summary scores did not differ significantly between age groups, and no significant differences were observed in any diabetes‐specific HRQoL domains. These findings suggest that the lower HRQoL observed among adolescents may be primarily related to psychosocial rather than physical aspects of living with T1DM. These differences may reflect the increasing responsibility for diabetes self‐management, greater academic demands, and emotional and social challenges that accompany adolescence [18, 22, 33].

Lifestyle behaviors represented important factors related to HRQoL among adolescents and children with T1DM in this study. A considerable proportion of participants reported limited PA and challenges with dietary self‐management, including inconsistent carbohydrate counting and unhealthy eating patterns. Although girls tended to report lower levels of regular PA than boys, this difference was not statistically significant. Regular PA was independently associated with better HRQoL, highlighting its potential importance for adolescents and children with T1DM. Previous studies have shown that regular PA is associated with better glycemic control, psychosocial well‐being, and HRQoL among children with T1DM [39, 40]. Similarly, carbohydrate counting is an important component of diabetes self‐management and has been associated with improved glycemic management and self‐management skills [41, 42]. These findings highlight the importance of structured nutrition education, carbohydrate counting training, and promotion of regular PA as part of comprehensive pediatric diabetes care, while recognizing that behavioral and educational barriers may limit their implementation [35, 42, 43].

In the present study, male sex, regular PA, and consistent carbohydrate counting were independently associated with higher HRQoL. These findings are consistent with previous studies suggesting that lifestyle behaviors and effective self‐management may be related to improved perceived quality of life in pediatric diabetes populations [23, 33, 40]. However, these associations should be interpreted with caution because the cross‐sectional design does not allow causal conclusions. In this study, HbA1c was not significantly associated with HRQoL in the multivariable linear regression model. Although glycemic control is an important clinical indicator in diabetes management, previous studies have also reported inconsistent or non‐significant associations between HbA1c and HRQoL among children and adolescents with T1DM [22, 33]. The absence of a significant association between HbA1c and HRQoL in the present study may reflect the multifactorial nature of HRQoL, where psychosocial, behavioral, and family‐related factors may also contribute to patients' perceived quality of life [44, 45].

In the current study, parents consistently reported lower scores than children across all generic HRQoL domains, including physical, emotional, social, and school functioning, as well as the physical health summary, psychosocial health summary, and total HRQoL score. Although the difference in total PedsQL scores was relatively small and might have limited clinical significance, the consistent pattern across domains suggests that parents perceived a greater impact of diabetes on their children's daily functioning. Similar discrepancies between children's and parents' reports have been reported in pediatric chronic diseases and may reflect differences in perspectives, parental concerns regarding disease complications, and greater involvement in daily diabetes management [26, 46]. Therefore, both perspectives provide complementary information and should be considered when assessing HRQoL in children with T1DM.

In this study, school and emotional functioning were among the most affected HRQoL domains, particularly according to parents' reports. Children with T1DM may experience difficulties related to school attendance, diabetes management during school hours, fear of hypoglycemia, and maintaining concentration during daily activities [22, 23]. Emotional challenges related to treatment demands and concerns about diabetes complications have also been recognized as important contributors to reduced HRQoL [15, 16, 25]. These findings highlight the importance of school‐based support, including diabetes education for teachers and school staff, individualized care plans, access to glucose monitoring and hypoglycemia management, as well as effective communication among families, schools, and healthcare providers. Such approaches may help reduce barriers to learning and improve participation in school activities.

Gender differences in QoL were also observed, with girls reporting significantly lower HRQoL than boys in physical, emotional, and social functioning, as well as the physical health summary, psychosocial health summary, and total HRQoL score. In the diabetes‐specific module, girls also had lower scores for treatment barriers, diabetes symptoms, diabetes‐related worry, diabetes‐related communication, and treatment adherence, whereas school functioning did not differ significantly between sexes. Similar findings have been reported previously, with lower HRQoL among girls possibly related to differences in psychological well‐being, body image concerns, and disease‐related stress [24, 34, 47, 48, 49, 50, 51]. Sociocultural and developmental factors, particularly during adolescence, may also contribute to these differences. In southwest Iran, gender‐related expectations, differences in social participation, and family roles may influence how children and adolescents experience diabetes management. However, these factors were not directly assessed in this study and should therefore be considered possible explanations rather than established determinants.

Several contextual factors may influence HRQoL among children with T1DM. Family support, parental education, access to healthcare resources, and opportunities for healthy lifestyle behaviors may affect disease management and psychosocial adaptation [23, 39, 40, 52, 53, 54, 55]. These findings indicate that psychosocial and behavioral factors influence HRQoL in pediatric T1DM in addition to metabolic management. Comprehensive diabetes care should therefore incorporate routine HRQoL assessment, psychosocial support, self‐management education, promotion of regular PA, support for carbohydrate counting, and family‐centered interventions, with particular attention to school‐related difficulties and subgroups requiring additional support, such as adolescent girls [16, 35, 56, 57, 58, 59, 60].

This study has several strengths, including the assessment of HRQoL from both children's and parents' perspectives and the use of validated Persian versions of the PedsQL instruments. The study also contributes evidence from a middle‐income Middle Eastern setting, where data on HRQoL among children with T1DM remain limited. However, several limitations should be considered. The cross‐sectional design prevents causal inference, and the single‐center recruitment using consecutive sampling may limit generalizability. In addition, HRQoL and lifestyle behaviors were assessed using self‐reported measures, which may be affected by recall and social desirability bias. Some potentially relevant factors, such as psychological status and family support, were not assessed. Future longitudinal, multicenter studies are warranted to better clarify the dynamic relationship between glycemic control, self‐management behaviors, and quality of life over time.

## 5 Conclusion

Children and adolescents with T1DM in this study had the lowest HRQoL scores in school functioning. Parents consistently perceived a greater impact of diabetes on their children's quality of life than the children themselves. Girls reported lower HRQoL than boys across both generic and diabetes‐specific domains, whereas younger children had better HRQoL than adolescents in several generic HRQoL domains. Regular PA and carbohydrate counting were associated with better HRQoL. These findings suggest that pediatric diabetes care should extend beyond glycaemic control to include routine HRQoL assessment, psychosocial support, self‐management education, healthy lifestyle promotion, and family‐centered care, with particular attention to adolescents and girls, who appear to be more vulnerable to poorer HRQoL.

## Author Contributions

**Solmaz Heidari:** conceptualization, methodology, supervision, writing – review and editing, resources, project administration. **Majid Aminzadeh:** conceptualization, methodology, supervision, writing – review and editing, resources, project administration. **Ronak Badiei:** investigation, data curation, writing – review and editing. **Mahtab Tamimi:** writing – review and editing, methodology. **Shooka Mohammadi:** formal analysis, writing – original draft, writing – review and editing.

## Funding

The authors have nothing to report.

## Ethics Statement

The Medical Ethics Committee of Ahvaz Jundishapur University of Medical Sciences (AJUMS) granted ethical approval for this study (No. IR.AJUMS.HGOLESTAN.REC.1403.064).

## Conflicts of Interest

The authors declare no conflicts of interest.

## Transparency Statement

Majid Aminzadeh affirms that this manuscript is an honest, accurate, and transparent account of the study being reported; no important aspects of the study have been omitted; any discrepancies from the study as planned have been explained.

## Data Availability

The data that support the findings of this study are available from the corresponding author upon reasonable request.
